# Dual localized mitochondrial and nuclear proteins as gene expression regulators in plants?

**DOI:** 10.3389/fpls.2012.00221

**Published:** 2012-09-25

**Authors:** Anne-Marie Duchêne, Philippe Giegé

**Affiliations:** Institut de Biologie Moléculaire des Plantes du Centre National de la Recherche Scientifique, University of StrasbourgStrasbourg, France

**Keywords:** mitochondria, nucleus, gene expression regulation, retrograde signaling, dual targeting, re-localization

## Abstract

Mitochondria heavily depend on the coordinated expression of both mitochondrial and nuclear genomes because some of their most significant activities are held by multi-subunit complexes composed of both mitochondrial and nuclear encoded proteins. Thus, precise communication and signaling pathways are believed to exist between the two compartments. Proteins dual localized to both mitochondria and the nucleus make excellent candidates for a potential involvement in the envisaged communication. Here, we review the identified instances of dual localized nucleo-mitochondrial proteins with an emphasis on plant proteins and discuss their functions, which are seemingly mostly related to gene expression regulation. We discuss whether dual localization could be achieved by dual targeting and/or by re-localization and try to apprehend the signals required for the respective processes. Finally, we propose that in some instances, dual localized mitochondrial and nuclear proteins might act as retrograde signaling molecules for mitochondrial biogenesis.

## INTRODUCTION

Mitochondria have arisen from the symbiosis of an α-proteobacteria with the archeal-like ancestor of eukaryotes (Gray, 1992; Lang et al., 1997; Gross and Bhattacharya, 2009). During evolution, massive gene transfer has occurred from the symbiont to its host cell (Gray, 1999). However, reverse genetic material transfer has also taken place as exemplified by the presence of nuclear originating retrotransposon-like sequences in mitochondrial genomes of higher plants (Unseld et al., 1997). Nevertheless, modern mitochondrial genomes are all extremely reduced as compared to their bacterial ancestor. They only encode approximately between 10 and 100 protein coding genes (Burger et al., 2003), thus merely a small fraction of the mitochondrial proteome, e.g., composed of an estimated 2000–3000 proteins in higher plants (Millar et al., 2005). Most of mitochondrial proteins are thus encoded in the nucleus, expressed in the cytosol and imported into mitochondria by a number of established pathways (Lithgow and Schneider, 2010). As a power station in eukaryotic cells, mitochondria perform key metabolic processes, in particular oxidative phosphorylation implemented by respiratory complexes. Interestingly, these essential multi-subunit complexes are composed of both mitochondrial encoded and nuclear encoded mitochondrial proteins. It is thus evident that precise gene expression coordination and communication pathways from mitochondria to the nucleus must exist to allow the biogenesis of respiratory complexes and proper cell function. In plants, retrograde regulatory pathways from chloroplasts to the nucleus have been well documented. They include control by epistasy of synthesis and a pathway involving tetrapyrroles and plastid gene expression (Pogson et al., 2008). In mitochondria, considerably less information is available, although it has been suggested that reactive oxygen species might be involved in retrograde signaling to the nucleus (Rhoads and Subbaiah, 2007). Nonetheless, proteins that would be localized to both mitochondria and the nucleus would also make very good candidates for an involvement is such communication pathways (Krause and Krupinska, 2009). It is now becoming increasingly evident that organellar protein multi-localization is a much more widespread phenomenon than previously thought. It has in particular been very often found for proteins dual targeted to both mitochondria and chloroplasts (Carrie et al., 2009a). For some protein families this dual targeting even seems to be the normal situation (Duchêne et al., 2005). However, it is also emerging that dual localization often takes place as well between organellar proteins and the nucleus (Krause and Krupinska, 2009), in particular as exemplified by the growing list of proteins found in both plant mitochondria and the nucleus. Here, we discuss the function of the identified nucleo-mitochondrial proteins and examine possible dual localization mechanisms and signaling pathways.

## UAL TARGETING IS WIDESPREAD IN PLANTS

Dual-targeted proteins are defined as proteins encoded by a single gene and localized in two cellular compartments. In consequence, the two isoforms have identical sequences in most of their length but can slightly differ in their extremities, due to the presence of targeting sequences. In plant cells, the first dual-targeted protein was identified in 1995. It is a pea glutathione reductase found in both mitochondria and chloroplasts. Since this discovery, numerous proteins were identified to be dual targeted. The vast majority, about one hundred of them, are plastidial and mitochondrial proteins (Carrie and Small, 2012). A dozen are mitochondrial and peroxisomal (Michaud and Duchêne, 2012), and an additional 10 proteins are nuclear and plastidial (Krause and Krupinska, 2009). Finally, some proteins have been found in mitochondria and plasma membrane, in mitochondria and endoplasmic reticulum (ER), in plastids and ER, or even in mitochondria, chloroplasts, and the cytosol (Michaud and Duchêne, 2012).

The mechanisms allowing dual localization are multiple. Most of the dual mitochondrial–plastidial proteins have ambiguous targeting signals in their N-terminal extremities that are recognized by both mitochondrial and chloroplastic import apparatus. In contrast, distinct sorting sequences were identified in some mitochondria–peroxisome proteins, i.e., a N-terminal mitochondrial targeting sequence (MTS) and a C-terminal peroxisomal targeting signal, respectively (Michalecka et al., 2003; Carrie et al., 2009a). Post-translational modifications were also shown to allow dual targeting, e.g., farnesylation was proposed to control the localization of AtIPT3 to either plastids or the nucleus (Galichet et al., 2008). Re-localization mechanisms have also been suggested. For example, protein transport from the ER to plastids has been proposed for AtCaAH1, and post-translational modifications clearly influence CAH1 trafficking (Buren et al., 2011).

## NUCLEO-MITOCHONDRIAL PROTEINS IN EUKARYOTES

The dual localization of proteins to mitochondria and the nucleus is also a common phenomenon and has been described in several instances. In many eukaryotes, it has become obvious that a higher as previously thought number of mitochondrial proteins have more than one localization. In particular, it has been shown that, up to one third of the mitochondrial proteome of yeast is composed of dual localized proteins (an estimated 316 out of 801 proteins; Ben-Menachem et al., 2011). Among them, a significant proportion of proteins could be localized to both mitochondrial and the nucleus (Yogev and Pines, 2011). Examples of nucleo-mitochondrial proteins include LRPPRC, a protein belonging to the pentatricopeptide repeat protein family (Lurin et al., 2004; Schmitz-Linneweber and Small, 2008). In human, a mutation in this protein was found to be responsible for the French-Canadian type of Leigh syndrome (Mootha et al., 2003; Xu et al., 2004). In mitochondria, LRPPRC is necessary for polyadenylation and coordination of translation of mitochondrial messengers (Ruzzenente et al., 2012). However, this protein was first described as part of a ribonucleoprotein complex accountable for the shuttling of mRNAs from the nucleus to the cytosol (Mili and Pinol-Roma, 2003). In addition, LRPPRC has been suggested to be a cofactor of the eukaryotic translation initiation factor 4E (Topisirovic et al., 2009). Finally, in the nucleus, this protein was proposed to regulate the expression of nuclear genes involved in mitochondrial biogenesis (Cooper et al., 2006). Other instances of nucleo-mitochondrial proteins include, e.g., ELAC2, a protein responsible for RNase Z activity, that removes 3′ trailer sequences of tRNA precursors, in both mitochondria and the nucleus in human (Rossmanith, 2011). TERT, the catalytic subunit of telomerase in the nucleus is exported from the nucleus during oxidative stress and imported into mitochondria where it protects the mitochondrial genome from reactive oxygen species (Ahmed et al., 2008). Finally, the nuclear transcription factor p53 involved in apoptosis through the activation or repression of pro- or anti-apoptotic genes, respectively, localizes to mitochondria during stress conditions. In mitochondria it uses its DNA binding domain to form an inhibitory complex with BclXL and Bcl2 (Mihara et al., 2003). Interestingly, all these examples of proteins found in both mitochondria and the nucleus appear to be involved in the control of gene expression or in post-transcriptional processes.

## OCCURRENCE OF NUCLEO-MITOCHONDRIAL PROTEINS IN PLANTS

Contrary to other eukaryotes such a yeast or human, very few instances of nucleo-mitochondrial proteins have been described in plants. Here we review the known examples of these dual localized proteins and examine their function (**Table 1**).

**Table 1:** Proteins identified in both mitochondria and the nucleus in plants.

Protein	Mito loc	Nuc loc	Function in Mito	Function in Nuc	Reference
AtTRZ3	GFP	GFP	RNase Z	RNase Z	Canino et al. (2009)
AtLigl	GFP	GFP	DNA repair?	DNA repair	Sunderland et al. (2006)
PsTrxol	GFP,W, IG	GFP,W, IG	Thioredoxin	Oxidation protection of DNA?	Marti et al. (2009)
DHFR	IG, Act	IG?	Dihydrofolate reductase	Unknown	Luo et al. (1997)
AtAPL	GFP	GFP	Unknown	Transcription factor	Carrie et al. (2009b)
AtPNMl	GFP,W, IG	GFP,W, IG, IC	Translation?	Gene expression negative regulator	Hammani et al. (2011a)

*At and Ps represent proteins identified in Arabidopis thaliana and Pisum sativum, respectively. “Mito loc”; and “Nuc loc”; indicate the methods used to localize proteins in mitochondria and the nucleus, respectively. GFP stands for green fluorescent protein fusion visualized by confocal microscopy;W stands for western blot analysis on purified cell fractions; IG stands for immunogold labeling; Act stands for activity assays with purified cell fractions, and IC stand for immunocytology. Envisaged or characterized functions in mitochondria and the nucleus are indicated. Question marks represent speculative location or functions in the respective compartments. It should be noted that many of the dual localizations described here were based on GFP fusion experiments. This technology sometimes leads to unspecific nuclear localizations when the fusion proteins are not large enough to be excluded from passive diffusion through nuclear pores (Seibel et al., 2007). Thus, occurrences of dual localizations of small proteins that were not confirmed by independent methods should be considered with caution.*

Similar to human, RNase Z is present in different copies in plants. Out of the four RNase Z proteins found in *Arabidopsis*, green fluorescent protein (GFP) fusion experiments showed that one of them has a localization restricted to mitochondria, whereas another one, AtTRZ3, appears to be dual localized to both mitochondria and the nucleus (Canino et al., 2009). This protein seems to be the only nuclear RNase Z in *Arabidopsis*. However, its gene was found to be non-essential. This hints that dual nuclear localization of other RNase Z isoforms in plants might have been overlooked.

Isoforms of the *Arabidopsis* DNA ligase 1 protein (AtLig1) have been found to localize to both mitochondria and the nucleus by GFP fusion experiments and confocal microscopy (Sunderland et al., 2006). In the two compartments, AtLig1 is expected to be involved in the final step of DNA repair processes (Tomkinson and Mackey, 1998). In the nucleus, this protein was indeed shown to play a crucial role in both DNA replication and excision repair pathways (Taylor et al., 1998). However, in mitochondria its precise molecular function has not yet been confirmed.

PsTrxo1, an isoform of thioredoxin in pea, was found in both mitochondria and nuclei by western blot analysis on purified cellular fractions, immunogold labeling as well as GFP fusions experiments (Marti et al., 2009). Thioredoxins are ubiquitous small proteins involved in the reduction of disulfide bonds of proteins. In mitochondria, PsTrxo1 is able to activate AOX and is proposed to reduce a number of predicted mitochondrial targets (Marti et al., 2009). Contrary to some mammalian thioredoxins that accumulate in the nucleus under stress conditions (Wei et al., 2000), PsTrxo1 is found in the nucleus in normal conditions (Marti et al., 2009). Its function at this location could be related to transcriptional regulation through oxidation protection of heterochromatin as proposed for the mammalian PRDX5 (Kropotov et al., 2006).

The plant mitochondrial dihydrofolate reductase (also found in plastids) was proposed to be present in plant nuclei as well because it was detected in the nucleolus of carrot by immunogold labeling (Luo et al., 1997; Krause and Krupinska, 2009). However, other studies in pea leaves clearly fail to identify dihydrofolate reductase activity in the nucleus (Neuburger et al., 1996). The occurrence of this protein as a nucleo-mitochondrial protein thus remains uncertain.

In *Arabidopsis*, the “altered phloem development”; (AtAPL) transcription factor was found dual localized to mitochondria and the nucleus by a high throughput GFP fusion approach (Carrie et al., 2009b). This MYB coiled-coil type transcription factor was shown to regulate vascular tissue identity in *Arabidopsis* through the regulation of nuclear genes (Bonke et al., 2003), whereas its mitochondrial function is unknown.

Finally, AtPNM1, an *Arabidopsis* pentatricopeptide repeat protein, was identified in both mitochondria and the nucleus by immunodetections on purified cell fractions, by immunocytology, by immunogold labeling, and by GFP fusion experiments (Hammani et al., 2011a). The function of this protein in mitochondria is unclear although it could be related to translation because PNM1 is associated with mitochondrial polysomes. In the nucleus, it interacts with a nucleosome assembly protein as well as with a TCP transcription factor (Hammani et al., 2011a). This class of protein was proposed (Welchen and Gonzalez, 2006) and shown (Giraud et al., 2010) to be involved in the coordinated expression of nuclear encoded mitochondrial proteins. Hence, mutants expressing a version of PNM1 unable to localize to the nucleus revealed that some nuclear genes encoding mitochondrial proteins had increased mRNA levels in the absence of PNM1. This has thus suggested that in the nucleus, PNM1 might act as a negative regulator for the expression of nuclear encoded mitochondrial proteins (Hammani et al., 2011a ,2011b).

Nevertheless, it appears that most plant nucleo-mitochondrial proteins, similar to their other eukaryotic counterparts, seem to be involved in the control of gene expression. These proteins therefore make very good candidates for the regulation of gene expression coordination between mitochondria and the nucleus (Giegé et al., 2005; Giraud et al., 2010) required for mitochondrial biogenesis.

## HOW IS NUCLEO-MITOCHONDRIAL DUAL LOCALIZATION ACHIEVED?

For AtTRZ3, AtLig1, PsTrxo1, AtAPL, and AtPNM1, the dual nucleo-mitochondrial localization appears to be clearly established. These five proteins all have two distinct putative targeting signals, a MTS and a NLS. The two distinct sorting sequences MTS and NLS often compete with each other, leading to the dominance of one sorting signal over the second one. The main strategies for subcellular targeting appear to be the synthesis of proteins isoforms, either by alternative transcription start, alternative splicing, or alternative translation initiation.

**FIGURE 1:**
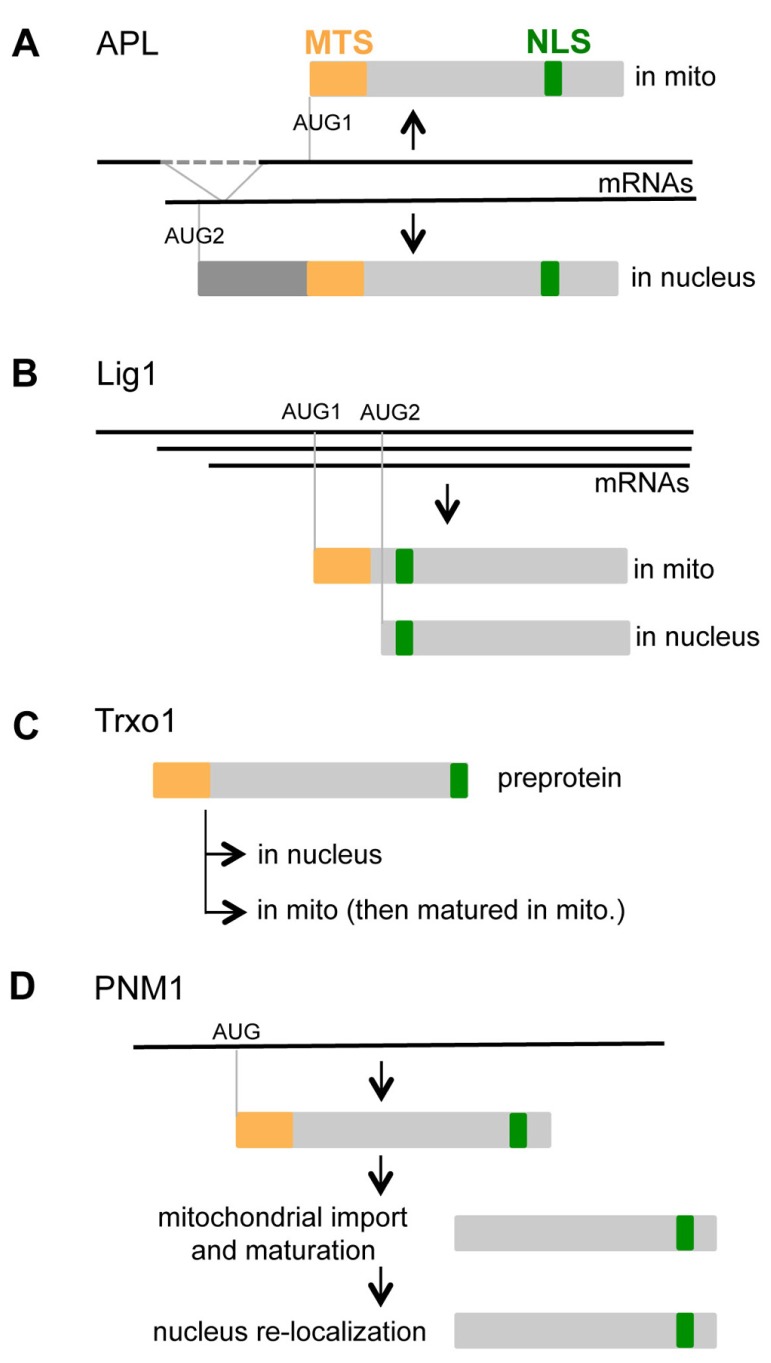
**Nucleo-mitochondrial dual targeting of proteins is controlled by different mechanisms.** **(A)** Alternative splicing of APL generates two transcripts that code for different proteins. **(B)** Alternative initiation of transcription and of translation controls the balance of mitochondrial and nuclear Lig1. **(C)** The Trxo1 pre-protein is imported into both mitochondria and the nucleus, although the control of partition between the two organelles is not yet understood. **(D)** The nuclear localization of PNM1 can be explained by re-localization of the mitochondrial mature protein into the nucleus.

Alternative splicing of AtAPL generates two transcripts with different 5′ extremities. Both encode protein isoforms with the MTS and NLS sequences (**Figure 1**). The first transcript codes for a protein of 293 aminoacids (aa) with a MTS at its N-terminal extremity. The second transcript codes for a larger protein (358 aa) with a N-terminal extension in front of its MTS sequence. Bonke et al. (2003) have demonstrated that GFP fused to the N-terminus of the larger protein is targeted to the nucleus. Carrie et al. (2009b) have shown that GFP fused to the C-terminus of the shorter protein is imported into mitochondria, and no signal is observed in the nucleus. Mitochondrial import appears dominant over nuclear targeting, suggesting that the MTS is dominant over the NLS. It has been proposed that the N-terminal extension in the larger protein masks the MTS, thus allowing targeting to nucleus.

For AtLig1, alternative transcription initiation yields three mRNA with different 5′ UTR. However, in that case, the coding sequence is identical for the three transcripts. Two in-frame AUG codons are present on these transcripts (**Figure 1**), and a mechanism of alternative translation initiation has been proposed. In a cell-free coupled transcription/translation system, translation initiation occurs at the first and the second AUG (half for each). Both the nucleotides context around the alternative start codons and the length of the 5′ UTR influence translation initiation efficiency (Sunderland et al., 2006). Fusions with GFP have revealed different targeting of the two protein isoforms. Translation initiation from the first start codon produces a mitochondrial isoform, on the other hand initiation from the second start codon produces a nuclear isoform. The larger protein contains both the MTS and NLS sequences, and the MTS appears dominant over the NLS. The smaller protein only containing the NLS is targeted to the nucleus.

Complementary approaches have indicated that the pea Trxo1 is dual localized in the nucleus and mitochondria (Marti et al., 2009). The molecular weight (MW) of the nuclear isoform is consistent with the MW of the pre-protein, and the MW of the mitochondrial isoform is in accordance with the maturation of the pre-protein after import. The pre-protein is thus imported into both mitochondria and nucleus, although the mechanism of partition between the two compartments is not known.

One unique transcript was detected for PNM1, and no in-frame AUG codon was found close to the initiation codon (Hammani et al., 2011a). Surprisingly, the isoforms of PNM1 detected in both compartments have a MW corresponding to the mature protein. As no alternative translation initiation is suspected, it is tempting to speculate that PNM1 is imported into mitochondria, matured, then partially re-localized into the nucleus (Hammani et al., 2011b). Such a mitochondria to nucleus shuttle had never been shown in plant but had been proposed for yeast fumarase (Singh and Gupta, 2006). Similarly, chloroplast to nucleus shuttling has been proposed for transcription factors such as pBrp or Tsip1 (Lagrange et al., 2003; Ham et al., 2006). It is thus imaginable that proteins such as PNM1 might relocate from the mitochondrial surface to the nucleus through a similar mechanism.

## ENVISAGED NUCLEO-MITOCHONDRIAL SIGNALING PATHWAYS

Signaling pathways between mitochondria and the nucleus include both anterograde and retrograde regulations. Anterograde control of the nucleus over mitochondria is extensively documented with the incidence of the proteins responsible for mitochondrial gene expression, which are almost all encoded in the nucleus and subsequently targeted to mitochondria, thus enabling a precise control of the nucleus on mitochondrial function (Giegé and Brennicke, 2001).

On the other hand, little information is available on plant mitochondrial retrograde regulation (Pogson et al., 2008). Reactive oxygen species (ROS), that are often regarded as markers of mitochondrial oxidative stress, have been proposed to be involved in mitochondrial retrograde signaling to the nucleus (Rhoads and Subbaiah, 2007), similar to plastidial ones (Nott et al., 2006). In this process, sensors such as ROS scavenging proteins, could participate in the early steps of a signaling pathway that would ultimately lead to gene expression changes in the nucleus (Gray et al., 2004; Giegé, 2007). Other potential participants in mitochondrial retrograde regulation could be proteins belonging to “two-components”; pathways. This type of signaling pathways that was until recently believed to be exclusively prokaryotic has been found in plants (Grefen and Harter, 2004). It implicates at least two proteins: a signal sensing histidine kinase and a response regulator that elicits the output response. ARR2, a response regulator protein primarily expressed in pollen is localized in the nucleus where it functions as a transcription factor, e.g., ARR2 regulates *in vivo* the promoter region of a mitochondrial complex I subunit nuclear gene (Lohrmann et al., 2001). Finally, the occurrence of proteins such as PNM1 that seem to be released from mitochondria in order to be re-localized to the nucleus suggests that some nucleo-mitochondrial proteins might act directly as retrograde signaling molecules for the coordination of gene expression in both mitochondria and the nucleus, as required for proper mitochondrial biogenesis.

## CONCLUDING REMARKS

The growing list of proteins dual localized to mitochondria and the nucleus in plants, but also in other eukaryotes, shows that many of these proteins have a role in genome maintenance or in gene expression regulation, similar to what is also observed for nucleo-plastidial proteins (Desveaux et al., 2005). It has been hypothesized that the rationale for the occurrence of these proteins in two compartments might be for the sequestration of proteins in one compartment until specific environmental or developmental cues require their function in the other (Krause and Krupinska, 2009). Alternatively, proteins might be dual targeted to mitochondria and the nucleus to perform the same functions for the respective genomes expression in the two compartments and thus to act as direct effectors of gene expression coordination. Nonetheless, it is also imaginable that some nucleo-mitochondrial proteins might act as signaling molecules from mitochondria to the nucleus. Overall the analysis of the identified instances of plant proteins localized to both mitochondria and the nucleus, suggests that these proteins have evolved different strategies to achieve dual localization, which enables them to act as regulators for the coordinated expression of the mitochondrial and nuclear genomes.

## Conflict of Interest Statement

The authors declare that the research was conducted in the absence of any commercial or financial relationships that could be construed as a potential conflict of interest.
